# Factors associated with blood pressure check-up during pregnancy among women of reproductive age in Tanzania: an analysis of data from 2015—16 Tanzania Demographic and Health Survey and Malaria Indicators Survey

**DOI:** 10.1186/s12884-021-03963-7

**Published:** 2021-06-30

**Authors:** Fabiola V. Moshi, Maximilian Tungaraza

**Affiliations:** 1 Department of Nursing Management and Education, School of Nursing and Public Health, The University of Dodoma, P. O. Box 259, Dodoma, Tanzania; 2 Department of Clinical Nursing, School of Nursing and Public Health, The University of Dodoma, P. O. Box 259, Dodoma, Tanzania

**Keywords:** Blood pressure, Check-ups, Pregnant women

## Abstract

**Background:**

Hypertensive Disorder of Pregnancy (HDP) is one of the leading causes of maternal mortality and morbidity amongst pregnant women in the world. Blood pressure check-ups during pregnancy are one of the strategies used to identify hypertensive disorders, hence timely management. Little is known about the factors associated with blood pressure check-ups in Tanzania.

**Method:**

The study used data from 2015—16 Tanzania Demographic and Health Survey and Malaria Indicators Survey (2015—16 TDHS—MIS). A total of 6924 women of active reproductive age from 15 to 49 were included in the analysis. Both univariate and multiple regression analyses were used to determine the association between early antenatal booking and maternal services utilization.

**Results:**

The prevalence of blood pressure checkups during pregnancy was 72.17% at 95% confidence interval of 71.1–73.2%. Factors associated with uptake of blood pressure check-ups were; timely antenatal booking, AOR = 1.496, CI = 1.297–1.726, *p* < 0.001, late booking was a reference population, age group [> 34 years, (AOR = 1.518, CI = 1.149–2.006, *p* = 0.003)] with < 20 years used as a reference population, wealth index [middle income, (AOR = 1.215, CI = 1.053–1.468, *p* = 0.008) and rich, (AOR = 2.270, CI = 1.907–2.702, *p* < 0.001)] reference population being poor; education level [primary education, (AOR = 1.275, CI = 1.107–1.468, *p* = 0.001); secondary education, (AOR = 2.163, CI = 1.688–2.774, *p* < 0.001) and higher education, (AOR = 9.929, CI = 1.355–72.76, *p* = 0.024)] reference population being no formal education; parity [para 2–4, (AOR = 1.190, CI = 1.003–1.412, *p* = 0.046) with para one used as a reference population and zones [Unguja Island, (AOR = 3.934, CI = 1.568–9.871, *p* = 0.004), Pemba Island, (AOR = 5.308, CI = 1.808–15.58, *p* = 0.002)] and Mainland Urban being the reference population.

**Conclusion:**

The study revealed that rural dwelling pregnant women had higher chance of not getting their BP checked. It was also revealed that maternal age, education level, place of residence, wealth index and timing of ANC services were significantly associated with blood pressure check-ups. The study recommends the need to explore significant factors associated with utilization of available free reproductive health services across all public health facilities. It also recommends the need to address prioritized intensive awareness programs and behavioral change interventions on the significance of BP check-ups among pregnant women of reproductive age.

## Background

Maternal mortality is inadmissibly high worldwide. In 2017, about 295,000 women died during pregnancy and childbirth. About 94% of these deaths occurred in low-resource settings and most of them could have been prevented [1]. Sub-Saharan countries are reported to have 533 maternal deaths per 100,000 live births, which are equal to 200,000 maternal deaths yearly. The SSA alone accounts for the two thirds of all global maternal deaths per year [2]. In Tanzania, about 556 maternal deaths per 100,000 live births occurred [3]. Maternal deaths occur due to both direct and indirect causes. The direct causes of maternal deaths include postpartum haemorrhage, hypertensive disorders, puerperal sepsis, unsafe abortion and obstructed labour [4].

Globally, HDP is one of the leading direct causes of maternal mortality and morbidity amongst pregnant women [5, 6] as it accounts for nearly 12% of the global maternal deaths [5]. Hypertension in pregnancy is a condition in which systolic blood pressure (SBP) measures ≥ 140 mmHg and/or diastolic blood pressure (DBP) of ≥ 90 mmHg which is confirmed when blood pressure is measured within 4 h apart. HDP encompasses a variety of disorders which include pre-existing hypertension, gestational hypertension, preeclampsia/eclampsia, and superimposed hypertension [6]. Pre-eclampsia and eclampsia cause the most serious consequences to the mother and baby [7]. These conditions are associated with vasospasm, pathologic vascular lesions in multiple organ systems, increased platelet activation and result in derangement of coagulation system in the small blood vasculature. A study done in Turkey found out that hypertensive disorders constituted the third most frequent cause among all causes and the second among direct causes of maternal deaths [8].

Tanzania ranked the 4^th^ which was the highest number of maternal deaths in sub-Saharan Africa and the 6^th^ which was the highest in the world [9]. Hypertensive disorders, especially eclampsia, contributed about 19% of maternal deaths and it was reported the 2^nd^ after postpartum hemorrhage in Tanzania [10]. The incidence of preeclampsia ranging from 1.8 to 16.7% was estimated to be seven times higher in developing countries than in developed countries [7].

Blood pressure (BP) check-ups during pregnancy comprise one of the strategies to identify hypertensive disorders, hence timely management [11]. However, the check-ups depend on adequate and timely antenatal care (ANC) services utilization. ANC attendance offers the mother the opportunity to undergo blood pressure check-ups [12, 13]. Several studies have shown that the majority of women in sub-Saharan Africa book ANC services late [12–14]. Recent WHO guideline recommends the pregnant woman to undergo blood pressure check-ups for not less than eight times, whereby the first check-up takes place in the first trimester, 2 check-ups take place in the second trimester, and 5 check-ups take place in the third trimester [15]. Late ANC initiations deny pregnant women from meeting this goal, thus becoming difficult to detect and manage HDP timely [16]. According to TDHS 2015/16, about 49% of pregnant women did not complete the recommended ANC contacts, hence likely to miss the opportunity to check their blood pressure [17].

Potential associated factors were extracted from the literature and included maternal age, place of residence, education level, socioeconomic status, parity and timing of ANC services. Maternal age has been reported in some studies [18, 19]. Moreover, two studies done in Ethiopia found out that place of residence and education level of the woman contributed to status of ANC attendance for BP checkups [20, 21]. Educated women have greater awareness of the existence of ANC services and the benefits of using such services [22]. Furthermore, socioeconomic status of the woman was also a challenge. Several studies showed that good economic status of the woman enables her to complete the required number of BP check-ups [19, 22, 23]. Moreover, a systematic review study that included 74 studies found out that low parity was associated with adequate ANC service utilization [23]. Women with high parity tend to under-utilize ANC services, thus missing the opportunity to have their blood pressure adequately checked [24, 25].

However, little is known about the factors associated with blood pressure check-ups in Tanzania. The current study, therefore, set out to analyze the factors associated with blood pressure check-ups during pregnancy among women of reproductive age in Tanzania using the data from the 2015—16 Tanzania DHS and Malaria Indicators Survey.

## Methods

### Study area and period

The study was conducted in the United Republic of Tanzania from August 22^nd^, 2015, through February 14^th^, 2016. Tanzania is among the countries found in East Africa, It is the largest country that covers 940,000 square kilometers and 60,000 square kilometers constitute inland water [26]. The country lies south of the equator and shares the borders with eight countries namely Kenya and Uganda to the North; Rwanda, Burundi, the Democratic Republic of Congo, and Zambia to the West; and Malawi and Mozambique to the South.

### Study design

It was a national-based cross-sectional study which utilized the 2015—16 Tanzania Demographic and Health Survey and Malaria Indicator Survey (TDHS-MIS) dataset.

### Study population

All women of reproductive age (aged 15 to 49) comprised the study population. The study used individual file records (TZIR7BFL) with a total of 13,266 women who responded to the survey (97% response rate). The study included only women who remembered the timing for antenatal booking of their youngest child. Those who were unable to recall the timing and respond to the questions were removed from the analysis. A total of 6924 women who had birth within five years before the survey were included in the study.

### Sampling technique

Two stages of sampling were used to obtain a sample for urban and rural areas in Tanzania Mainland and Zanzibar. The total of 608 clusters was selected in the first stage whereas the total of 22 households was systematically selected in the second stage. The systematic selection of households from each cluster yielded a representative probability sample of 13,376 households for the 2015—16 TDHS—MIS. To enhance representativeness, Tanzania was divided into nine (9) geographic zones. Grouping the regions into zones was done in order to reduce sampling error by increasing the number of people in the denominator. The referred zones comprised Western zone (Tabora and Kigoma Region), Northern zone (Kilimanjaro, Tanga, and Arusha Region), Central zone (Dodoma, Singida and Manyara Region), Southern Highland zone (Iringa, Njombe, and Ruvuma Region), and Southern zone (Lindi and Mtwara Region). Other zones included South West Highland zone (Mbeya, Rukwa and Katavi Region), Lake zone (Kagera, Mwanza, Geita, Mara, Simiyu, and Shinyanga Region), Eastern zone (Dar es Salaam, Pwani, and Morogoro Region) and Zanzibar (Kaskazini Unguja, Kusini Unguja, Mjini Magharibi, Kaskazini Pemba and Kusini Pemba Region).

### Data collection tool

The 2015—16 TDHS—MIS used the household and individual questionnaires. These questionnaires based on the measure DHS standard and Malaria Indicator Survey questionnaires standards. They were adapted and modified in order to reflect the Tanzanian population. They were translated into Kiswahili which is the Tanzania’s national language. The data presented in this study were obtained from the individual questionnaires.

### Study variables

A. **Dependent Variable:** Blood pressure check-up
B. **Independent Variables:** Maternal age, place of residence, marital status, socioeconomic status (Wealth Index) and parity of the mother

### Data analysis

The data were analyzed using Statistical Package for Social Sciences (IBM SPSS version 20). The data analysis started by describing all study variables using the frequencies and percentages. The assessment of the association between the dependent variable and independent variables was done using the chi-square test. Finally, the binary logistic regression analysis (univariate and multivariate) to determine significant predictors for uptake of blood pressure check-up was performed during pregnancy.

## Results

With reference to Table 1, the study results indicated that 5113 (73.8%) study respondents resided in the rural setting of Tanzania, 4557 (65.8%) study respondents were aged 20 to 34, 4209 (60.8) study respondents had primary education and 5650 (86.1%) study respondents were married.

**Table 1:** Socio-demographic characteristics

**Variables**	**Frequency**	**Percent (%)**
**Place of residence**		
Urban	1811	26.2
Rural	5113	73.8
**Age group**		
Less than 20 years	541	7.8
20 to 34 years	4557	65.8
More than 34 years	1826	26.4
**Educational level**		
No education	1329	19.2
Primary education	4209	60.8
Secondary education	1326	19.2
Higher education	60	0.9
**Parity**		
Para one	1595	23
Para 2 – 4	3154	45.6
Para 5 +	2175	31.4
**Wealth index**		
Poor	2734	39.5
Middle	1363	19.7
Rich	2827	40.8
**Marital status**		
Never in union	441	6.4
Married	5650	86.1
Widow	119	1.7
Separated	714	10.3
**Respondent currently working**		
Not working	1498	21.6
Working	5426	78.4
**Mainland/Zanzibar**		
Mainland Urban	1618	23.4
Mainland Rural	4357	62.9
Unguja (Zanzibar Island)	594	8.6
Pemba (Pemba Island)	355	5.1

### Ever checked blood pressure during pregnancy

The prevalence of blood pressure checkups during pregnancy was 72.17% at 95% confidence interval of 71.1–73.2%.

### Relationship between women’s characteristics and ever checked blood pressure during pregnancy

With reference to Table 2, the study results indicate that the women’s characteristics which showed the significant relationship with ever checked for blood pressure were place of residence (*p* < 0.001), age group (*p* < 0.001), education level (*p* < 0.001), parity (*p* < 0.001), wealth index (*p* < 0.001) and zones (*p* < 0.001).

**Table 2:** Relationship between women’s characteristics and ever checked blood pressure during pregnancy

**Variables**	**Ever Checked**	**Never Checked**	**X2**	***P*-value**
	n (%)	n (%)		
**Place of residence**				
Urban	1593 (88)	218 (12)		
Rural	3404 (66.6)	1709 (33.4)	304.553	< 0.001
**Age group**				
15 – 19	340 (62.8)	201 (37.2)		
20 – 34	3319 (72.8)	1238 (27.20)		
35 – 49	1338 (73.3)	488 (26.7)	25.521	< 0.001
**Educational level**				
No education	823 (61.9)	506 (38.1)		
Primary education	2922 (69.4)	1287 (30.6)		
Secondary education	1193 (90.0)	133 (10)		
Higher education	59 (98.3)	1 (1.7)	314.868	< 0.001
**Parity**				
Para one	1165 (73)	430 (27)		
Para 2 – 4	2362 (74.9)	792 (25.1)		
Para 5 +	1470 (67.6)	705 (32.4)	34.964	< 0.001
**ANC** booking				
Late booking	3746 (70.2)	1592 (29.8)		
Early booking	1251 (78.9)	335 (21.1)	46.094	< 0.001
**Wealth index**				
Poor	1586 (58)	1148 (42)		
Middle	923 (67.7)	440 (32.3)		
Rich	2488 (88)	339 (12)	639.450	< 0.001
**Marital status**				
Never in union	325 (73.7)	116 (26.3)		
Married	4075 (72.1)	1575 (27.9)		
Widow	88 (73.9)	31 (26.1)		
Separated	509 (71.3)	205 (28.7)	0.981	0.806
**Mainland/Zanzibar**				
Mainland Urban	1405 (86.8)	213 (13.2)		
Mainland Rural	2676 (61.4)	1681 (38.6)		
Unguja (Zanzibar Island)	575 (96.8)	19 (3.2)		
Pemba (Pemba Island)	341 (96.1)	14 (3.9)	704.290	< 0.001

Having been adjusted for the confounders, the factors which influenced uptake of blood pressure check-ups during pregnancy included timing for antenatal booking within first 12 weeks, AOR = 1.496 at 95% CI = 1.297–1.726, *p* < 0.001, age group [more than 34 years, (AOR = 1.518 at 95% CI = 1.149–2.006, *p* = 0.003)], wealth index [middle income, (AOR = 1.215 at 95% CI = 1.053–1.468, *p* = 0.008) and rich, (AOR = 2.270 at 95% CI = 1.907–2.702, *p* < 0.001)] as with being poor, education level [primary education, (AOR = 1.275 at 95% CI = 1.107–1.468, *p* = 0.001), secondary, (AOR = 2.163 at 95% CI = 1.688–2.774, *p* < 0.001) and higher, (AOR = 9.929 at 95% CI = 1.355–72.76, *p* = 0.024)] as with no formal education, parity [para 2–4, (AOR = 1.190 at 95% CI = 1.003–1.412, *p* = 0.046) and zones [Unguja Island, (AOR = 3.934 at 95% CI = 1.568–9.871, *p* = 0.004), Pemba Island, (AOR = 5.308 at 95% CI = 1.808–15.58, *p* = 0.002)] and Mainland Urban being the reference population (see Table 3).

**Table 3:** Factors associated with ever checked blood pressure during pregnancy

**Variable**	**OR**	**95%CI**		***P*-value**	**AOR**	**95%CI**		***P*-value**
		**Lower**	**Upper**			**Lower**	**Upper**	
**ANC** booking								
Late booking	1				1			
Early booking	1.587	1.388	1.815	< 0.001	1.496	1.297	1.726	< 0.001
**Age groups**								
Less than 20 years	1							
20 to 34 years	1.585	1.316	1.909	< 0.001	1.193	0.951	1.498	0.127
More than 34 years	1.621	1.323	1.985	< 0.001	1.518	1.149	2.006	0.003
**Place of residence**								
Urban	1							
Rural	0.273	0.234	0.318	< 0.001	1.044	0.388	2.81	0.932
**Wealth index**								
Poor	1				1			
Middle	1.518	1.325	1.741	< 0.001	1.215	1.053	1.402	0.008
Rich	5.312	4.634	6.09	< 0.001	2.27	1.907	2.702	< 0.001
**Educational level**								
No education	1				1			
Primary education	1.396	1.227	1.588	< 0.001	1.275	1.107	1.468	0.001
Secondary education	5.515	4.468	6.808	< 0.001	2.163	1.688	2.774	< 0.001
Higher education	36.275	5.01	262.6	< 0.001	9.929	1.355	72.76	0.024
**Parity**								
Para one	1							
Para 2—4	1.101	0.96	1.262	< 0.001	1.19	1.003	1.412	0.046
Para 5 +	0.77	0.667	0.887	< 0.001	0.958	0.775	1.185	0.694
**Mainland/Zanzibar**								
Mainland Urban	1				1			
Mainland Rural	0.241	0.206	0.282	< 0.001	0.459	0.168	1.25	0.128
Unguja (Zanzibar Island)	4.588	2.841	7.409	< 0.001	3.934	1.568	9.871	0.004
Pemba (Pemba Island)	3.693	2.123	6.423	< 0.001	5.308	1.808	15.58	0.002

## Discussion

This study used the data from the 2015—16 Tanzania Demographic Health Survey and Malaria Indicators Survey to define the factors associated with blood pressure check-ups among pregnant women of reproductive age. The study found out that more married women from the rural residency dominated the study. Nearly three-quarter of the pregnant women checked their blood pressure.

The study found out that the timing of ANC services increased the likelihood of blood pressure check-ups among pregnant women. The finding at hand agrees with the findings reported by other studies conducted elsewhere in sub-Saharan Africa. For instance, the referred studies indicated that the women who started their ANC services early during pregnancy were more likely to get their blood pressure checked [12–14].

This study also found out that the age of women determined the blood pressure check-ups during pregnancy. For example, the women in higher age group were more likely to have adequate BP check-ups than their colleagues. This finding was consistent with the findings of a study done in Rwanda. A population-based and cross sectional study indicated that older women were more likely to get their BP checked since they had adequate ANC service utilization [19]. However, Ali et al. [18] reported the mixed findings which indicated that the younger the woman is, the higher the likelihood to utilize ANC services, thus increasing the chances of getting their BP checked. Besides, the same study showed that the majority of older women i.e., aged between 25 and 30 or above had higher chances to check their blood pressure more frequently with regard to their frequent ANC services utilization. Socio-cultural, ethnicity and geographical differences could be the cause for observed differences.

This study also found out that the place of residence determined the likelihood of the women to get their BP checked during pregnancy. A cross-sectional study based on demographic and health survey and a systematic review and meta-analysis study, which was conducted in Ethiopia revealed similar findings [20, 21]. The referred study indicated that rural women lack exposure to health information concerning the importance of attending ANC clinic for BP check-ups. The rural women are also less likely to be empowered compared to urban women. This study further indicated that pregnant women residing in Tanzania Mainland had a small chance of getting their BP checked during pregnancy compared to those dwelling in Tanzania Zanzibar. However, the women of child bearing age from Pemba were more likely to use ANC services for BP check-ups compared to those from Unguja. The geographical differences and quality of health care services provided could be the reason.

The current study indicates that women with higher education were nine or more times likely to check their blood pressure during pregnancy. This finding reflected the findings reported in the reviewed literature which is based on African countries [21]. The educated women tend to have greater awareness of the existence of ANC services and the benefits of using such services [22]. On the contrary, Rurangirwa et al. [19] found out that education had no any statistical significance.

The current study also revealed that richer women were two or more times likely to get their blood pressure checked during pregnancy compared to their counterparts. This finding is supported by the findings by the study which was conducted in Indonesia. The researcher argued that the richer women were four times more likely to have their BP checked during pregnancy compared to the poorer women [22]. Although Okedo-Alex et al. [23] supported it, Rurangirwa et al. [19] argued against it. According to Rurangirwa et al. [19], having household assets (which are proxy to socioeconomic status) contributed nothing to the woman’s reinforcement in utilizing ANC clinics for BP check-ups and other related services. This could be due to women empowerment differences irrespective of the family wealth.

The current study further indicated that the higher the parity, the reduced the chances of the woman to get their blood pressure checked during pregnancy. This is attributed by the fact that high parity women tend to rely on their experiences from previous pregnancies, and thus not feeling the need for ANC services [23]. The finding in question is further supported by two studies which were conducted in China and Tanzania [24, 25].

## Conclusion

Blood pressure check-ups during pregnancy offer the opportunity for early detection of timely management of HDP. The study revealed that rural dwelling pregnant women had the higher chances of not getting their BP checked during pregnancy. It was also revealed that maternal age, education level, place of residence, wealth index and timing of ANC services were significantly associated with blood pressure check-ups. The study recommends the need to explore significant factors associated with utilization of the available free reproductive health services across all public health facilities. It also recommends the need to address prioritized intensive awareness programs and behavioral change interventions on the significance of BP check-ups among pregnant women of reproductive age.

## Data Availability

The data that support this analysis are available at https://dhsprogram.com/data/ subject to permission from MEASURE DHS.

## Abbreviations

ANC: Antenatal clinic
BP: Blood pressure
DHS: Demographic Health Survey
DBP: Diastolic blood pressure
HDP: Hypertensive disorder of pregnancy
SBP: Systolic blood pressure
TDHS-MIS: Tanzania Demographic and Health Survey and Malaria Indicators Survey
WHO: World Health Organization

## References

[1] WHO. Monitoring health for the SDGs. Αγαη. 2019;8. Available from: https://apps.who.int/iris/bitstream/handle/10665/324835/9789241565707-eng.pdf.

[2] UNICEF (2019). Trends in estimates of maternal mortality ratio (maternal deaths per 100,000 live births) 2000–2017.

[3] National Bureau of Statistics (2016). Tanzania demographic and health survey and malaria indicator survey (TDHS-MIS) 2015–16.

[4] WHO. Maternal mortality evidence brief. Matern Mortal. 2019;(1):1–4. Available from: https://apps.who.int/iris/bitstream/handle/10665/329886/WHO-RHR-19.20-eng.pdf?ua=1.

[5] Tessema GA, Tekeste A, Ayele TA (2015). Preeclampsia and associated factors among pregnant women attending antenatal care in Dessie referral hospital, Northeast Ethiopia: a hospital-based study. BMC Pregnancy Childbirth.

[6] Hinkosa L, Tamene A, Gebeyehu N (2020). Risk factors associated with hypertensive disorders in pregnancy in Nekemte referral hospital, from July 2015 to June 2017, Ethiopia: case-control study. BMC Pregnancy Childbirth.

[7] Osungbade KO, Ige OK (2011). Public health perspectives of preeclampsia in developing countries: implication for health system strengthening. J Pregnancy.

[8] Keskinkılıç B, Engin-Üstün Y, Sanisoğlu S, Şahin Uygur D, Keskin HL, Karaahmetoğlu S (2017). Maternal mortality due to hypertensive disorders in pregnancy, childbirth, and the puerperium between 2012 and 2015 in Turkey: a nation-based study. J Turk Ger Gynecol Assoc.

[9] World Health Organization. Trends in maternal mortality: 1990 to 2013: estimates by WHO, UNICEF, UNFPA, The World Bank and the United Nations Population Division. Geneva; 2014. http://apps.who.int/iris/bitstream/handle/10665/112682/9789241507226_eng.pdf;jsessionid=598DFEB33D786D8F7F4C920EDB1D3C7E?sequence=2.

[10] Pembe AB, Paulo C, D’mello BS, van Roosmalen J. Maternal mortality at Muhimbili National Hospital in Dar-es-Salaam, Tanzania in the year 2011. BMC Pregn Childb. 2014;14(1):1–7. 10.1186/1471-2393-14-320 PMC4174678 25217326

[11] Nathan HL, Duhig K, Hezelgrave NL, Chappell LC, Shennan AH (2015). Blood pressure measurement in pregnancy. R Coll Obstet Gynaecol.

[12] Mgata S, Maluka SO (2019). Factors for late initiation of antenatal care in Dar es Salaam, Tanzania: a qualitative study. BMC Pregnancy Childbirth.

[13] Ebonwu J, Mumbauer A, Uys M, Wainberg ML, Medina-Marino A (2018). Determinants of late antenatal care presentation in rural and peri-urban communities in South Africa: a cross-sectional study. PLoS One.

[14] Gulema H, Berhane Y (2017). Timing of first antenatal care visit and its associated factors among pregnant women attending public health facilities in Addis Ababa, Ethiopia. Ethiop J Health Sci.

[15] World Health Organization (WHO) (2018). WHO recommendations on antenatal care for a positive pregnancy experience: summary. World Health Organ.

[16] Alemu Y, Aragaw A (2018). Early initiations of first antenatal care visit and associated factor among mothers who gave birth in the last six months preceding birth in Bahir Dar Zuria Woreda North West Ethiopia. Reprod Health.

[17] MoHCDGEC (2015). Tanzania demographic and health survey and malaria indicator survey.

[18] Ali SA, Dero AA, Ali SA, Ali GB (2018). Factors affecting the utilization of antenatal care among pregnant women: a literature review. J Adv Nurs.

[19] Rurangirwa AA, Mogren I, Nyirazinyoye L, Ntaganira J, Krantz G (2017). Determinants of poor utilization of antenatal care services among recently delivered women in Rwanda; a population based study. BMC Pregnancy Childbirth.

[20] Tiruaynet K, Muchie KF (2019). Determinants of utilization of antenatal care services in Benishangul Gumuz Region, Western Ethiopia: a study based on demographic and health survey. BMC Pregnancy Childbirth.

[21] Id TT, Chojenta C, Smith R, Loxton D (2019). Factors affecting utilization of antenatal care in Ethiopia : a systematic review and meta- analysis.

[22] Efendi F, Chen CM, Kurniati A, Berliana SM (2017). Determinants of utilization of antenatal care services among adolescent girls and young women in Indonesia. Women Health.

[23] Okedo-Alex IN, Akamike IC, Ezeanosike OB, Uneke CJ (2019). Determinants of antenatal care utilisation in sub-Saharan Africa: a systematic review. BMJ Open.

[24] Gross K, Alba S, Glass TR, Schellenberg JA, Obrist B (2012). Timing of antenatal care for adolescent and adult pregnant women in south-eastern Tanzania.

[25] Zhao Q, Huang ZJ, Yang S, Pan J, Smith B, Xu B (2012). The utilization of antenatal care among rural-to-urban migrant women in Shanghai: a hospital-based cross-sectional study. BMC Public Health.

[26] NBS (1964). History, geography, and economy geography.

